# Right heart thrombus-in-transit in a patient with Evans syndrome: A case report

**DOI:** 10.1097/MD.0000000000027009

**Published:** 2021-08-20

**Authors:** Ya-Chen Yang, Yen-Yu Chen

**Affiliations:** a Kaohsiung Chang Gung Memorial Hospital Education Department, Kaohsiung, Taiwan; b Division of Thoracic and Cardiovascular Surgery, Department of Surgery, Kaohsiung Chang Gung Memorial Hospital and Chang Gung University College of Medicine, Kaohsiung, Taiwan.

**Keywords:** case report, Evans syndrome, right heart thrombus, venous thromboembolism

## Abstract

**Introduction:**

Right heart free-floating thrombus in the absence of structural heart disease or atrial fibrillation is rare. When it travels to the heart into the lung, called thrombus-in-transit, may cause cardiopulmonary collapse and sudden death. The clinical presentation varies from mild respiratory symptoms to sudden death; however, there are few clinical case reports of giant, free-floating thrombus in the right heart in an asymptomatic patient, and the optimal management options have not been established.

**Patient concerns:**

A 36-year-old Asian woman presented to the emergency department with complaints of worsening swelling of the left lower extremity over 12 hours.

**Diagnosis:**

Left leg deep vein thrombosis accompanied by an asymptomatic giant right atrial thrombus and pulmonary embolism with a rare autoimmune disease of Evans syndrome.

**Interventions:**

Emergent surgical thrombectomy under cardiopulmonary bypass for right atrial thrombus.

**Outcomes:**

The postoperative course was uneventful, and she was discharged on the eighth postoperative day with normal heart function and mild tricuspid regurgitation.

**Conclusion:**

An additional diagnostic workup in cases of deep vein thrombosis is necessary for the rapid diagnosis of right heart thrombus and pulmonary embolism without delay. This case report illustrates that early recognition of venous thromboembolism and emergent thrombectomy of right heart thrombus-in-transit is crucial to prevent mortality.

## 1 Introduction

Right heart thrombus (RHT) is uncommon and is generally detected by echocardiography in patients with venous thromboembolism (VTE). It usually represents a traveling clot originating from the leg to the lung, known as right heart thrombus-in-transit.^[1]^ Right heart thrombus-in-transit is considered a medical emergency with a high mortality rate and requires immediate treatment.^[2]^ Evans syndrome (ES) is an uncommon autoimmune disease defined by the combination of autoimmune hemolytic anemia (AIHA) and immune thrombocytopenia (ITP), diagnosed in only 0.7% to 3.7% of all patients with either AIHA or ITP.^[3]^ Several published studies have reported an increased risk of VTE in patients with AIHA or ITP; however, only a few studies have reported thrombotic complications in patients with ES. Here, we report a case of ES that presented with left leg deep vein thrombosis (DVT) accompanied by an asymptomatic giant right atrial thrombus and pulmonary embolism (PE). To our knowledge, this is the first report of right heart thrombus-in-transit in an ES patient.

## 2 Case report

A 36-year-old Asian woman presented to the emergency department with complaints of worsening swelling of the left lower extremity over 12 hours. She underwent cesarean section because of pre-term pre-mature rupture of the membrane 5 months prior and 1 episode of chest pain and dyspnea, accompanied by left leg swelling during the postpartum period, which resolved spontaneously without any intervention. Her medical history was ES diagnosed during the second trimester of the last pregnancy that was treated with an initial dose of 1 mg/kg/day of prednisolone for 4 weeks, with tapering by 5 mg weekly. She recently received low-dose prednisolone (<10 mg/day) for the treatment of thrombocytopenia. She also had a history of right immature ovarian teratoma that was treated with right salpingo-oophorectomy with omentectomy and chemotherapy 7 years prior. This patient was a non-smoker, non-drinker, and denied any family history of autoimmune diseases or clotting disorders.

Her vital signs upon arrival were as follows: body temperature of 36.3 °C; blood pressure of 165/116 mm Hg; heart rate of 111 beats/min; and respiratory rate of 20 breaths/min. She denied any chest pain or dyspnea. On physical examination, breathing sounds were clear to auscultation bilaterally, and no cardiac murmurs were heard. The abdomen was soft, without hepatosplenomegaly. Examination of the extremities revealed erythema, warmth, tenderness, and swelling of the entire left leg, which was 4 cm larger in diameter than the right leg. Laboratory studies showed white blood cell counts of 7300/uL, hemoglobin of 13.2 g/dL, hematocrit of 38.4%, platelet count of 42,000/uL, and D-dimer level of 26.47 mg/L. Her serum electrolyte, blood urea nitrogen, creatinine, aspartate aminotransferase, prothrombin time, and activated partial thromboplastin time were within normal limits. Electrocardiography demonstrated normal sinus rhythm without ST segment changes. Chest radiography revealed a left pleural effusion and a normal heart size. With symptoms suggestive of DVT, she was administered subcutaneous enoxaparin sodium 6000iu every 12 hours.

Extensive thrombosis from the left femoral vein to the common iliac vein was observed on contrast-enhanced computed tomography angiography of the lower extremities performed the following day. A Celect Platinum retrievable inferior vena cava filter (Cook Medical, Bloomington, IN) was placed for iliofemoral DVT. Transthoracic echocardiography revealed a large, mobile, homogeneous, right atrial mass, approximately 7 cm in length, prolapsing through the tricuspid valve during diastole. No thrombi were detected in the main pulmonary artery via transthoracic echocardiography, and the right ventricle was not enlarged with normal systolic function.

**Figure 1:**
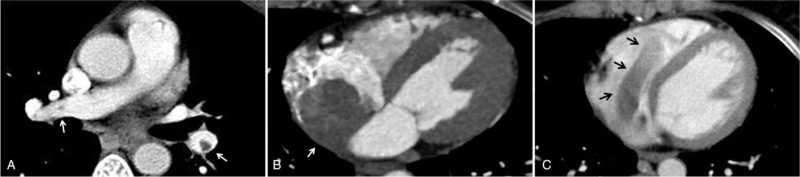
(A) Computed tomography angiogram showing multiple thrombi (arrow) in the right pulmonary artery and left interlobar artery. (B) Arrow indicates a large right atrial thrombus extending into the inferior vena cava. (C) Arrow indicates right atrial thrombus protruding into the right ventricle.

Subsequent computed tomography angiography of the chest revealed filling defects in the right pulmonary artery and left interlobar artery, consistent with thrombi (Fig. 1A). It also revealed a right atrial mass extending into the inferior vena cava and right ventricle (Fig. 1B, C).

**Figure 2:**
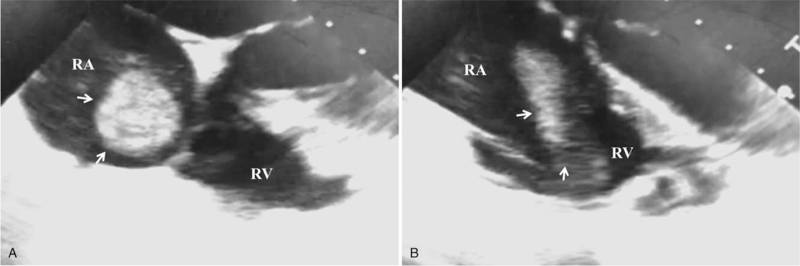
(A) Arrows indicate large right atrial mass via transesophageal echocardiography. (B) Mid-esophageal 4 chamber view shows the mass (arrow) protruding into the right ventricle. RA = right atrium, RV = right ventricle.

**Figure 3:**
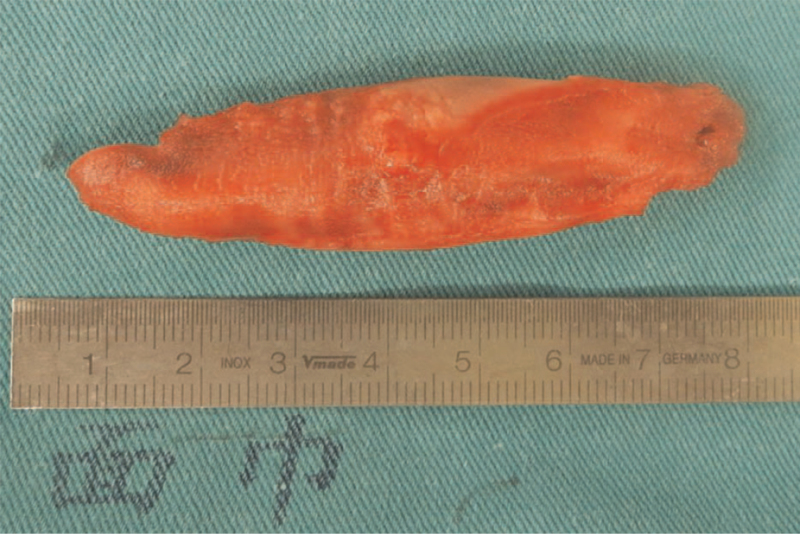
The organized thrombus excised from the right atrium at surgery.

Because it was thought to be a thrombus rather than myxoma, cardiology and cardiovascular surgeons were consulted regarding therapeutic options. Open thrombectomy and conservative thrombolytic therapy were discussed. The anticoagulant used for 7 days was not effective in resolving the right atrial mass, which was suggestive of an organized thrombus. Considering her underlying condition with thrombocytopenia and lower surgical risk, an open thrombectomy was suggested. The patient was scheduled to undergo surgery. Intraoperative transesophageal echocardiography revealed a large mass that moved into and out of the right ventricle (Fig. 2A, B). Surgical resection was performed via a lower mini-sternotomy. Cardiopulmonary bypass was established with bicaval drainage and right femoral artery perfusion. After a longitudinal right atriotomy, the mass was removed without cardioplegic arrest. No visible stalk or ulceration of the right atrium was observed. Histopathologic examination confirmed the mass as an organized thrombus that measured 7.0 × 1.9 × 1.0 cm (Fig. 3).

The postoperative course was uneventful, and her left leg swelling gradually improved. Further hypercoagulable workup revealed positive lupus antigen, decreased protein S level (25%), and normal antithrombin III, protein C, anticardiolipin antibody IgG/IgM, and anti-beta 2-glycoprotein-1 IgG. Transthoracic echocardiography before discharge showed mild residual tricuspid valve incompetence. The patient was discharged on the eighth postoperative day with anticoagulant therapy. The patient was treated with oral warfarin for 6 months postoperatively and had no further complications at the 6-months follow-up.

## 3 Discussion

RHT can be detected during routine echocardiographic examinations or in patients with PE. A large-scale population-based study reported the prevalence of RHT in 3.8% of patients with acute PE.^[4]^ Free-floating RHT is a medical emergency that carries a high mortality rate. In a series by Chartier et al,^[2]^ 21.1% of patients died on the first day of admission, with an in-hospital mortality rate of 44.7%. The mortality rates of PE patients with RHT are dependent on the severity of hypoxemia and hemodynamic status.^[2,5]^ The treatment options for RHT include anticoagulation therapy, thrombolytic therapy, and surgical embolectomy. The optimal therapy is still not standardized because of the lack of prospective randomized controlled studies. In a retrospective subgroup analysis of 123 patients with RHT conducted by Rose et al,^[6]^ surgery was associated with an increased risk of mortality compared with thrombolytic therapy. Although more recent prospective studies suggest that thrombolysis is a faster and readily available option to treat VTE, the side effects of bleeding and dissolved clot re-embolizing the lung might be problematic. Surgical embolectomy is still the preferred choice for emergent cases of large mobile thrombi, particularly in patients with bleeding disorders.^[2]^

ES is a rare autoimmune disease in which autoantibodies destroy red blood cells, platelets, and sometimes white blood cells. This leads to AIHA and ITP with a positive direct anti-human globulin test.^[7]^ Warm AIHA is the most common subtype in ES patients, and it has been reported that warm AIHA alone increases the risk of VTE, especially when this disease is active regardless of thrombocytopenia.^[8]^ In a series by Audia et al,^[8]^ 23% of adults with warm AIHA experienced at least 1 VTE during a median follow-up of 24.8 months. Several large-scale epidemiological studies^[9,10]^ have concluded that ITP patients have a higher risk of VTE than the general population. The presence of AIHA and ITP may explain the increased risk of VTE in ES patients. The pathogenesis of thrombosis in ES remains poorly defined, but several mechanisms have been proposed. Pro-inflammatory cytokines, cell-derived microparticles, and direct endothelial damage are believed to contribute to thrombus formation.^[11]^ High doses or long-term steroid use can also be an additional risk factor for thrombosis.

Our patient had a positive direct Coombs test for IgG and low-titer cold agglutinins (1:16), suggestive of warm AIHA, and ITP was diagnosed as persistent thrombocytopenia after exclusion of other possible causes. Although the patient had no high doses or long-term steroid use, a thromboembolic event occurred 8 months after the diagnosis of ES. The only possible thrombotic risk factor was ES, and she presented with extensive VTE in which blood clots travel to both lungs from deep veins in the left leg. It is important to scan the chest for RHT and PE once DVT is diagnosed. Due to the large size of the RHT, despite the absence of symptoms, we performed emergent surgical embolectomy to avoid sudden death from massive PE. This is a rare case of thromboembolic manifestations of ES.

## 4 Conclusion

Although right heart thrombus-in-transit is not rare, it requires emergent management due to the high risk of mortality and must be sought in all patients with DVT. Echocardiography is the choice for the detection of RHT, and computed tomography pulmonary angiography can be used for the diagnosis of PE. Additionally, ES is an autoimmune disease that may increase the risk of VTE. Due to the rarity of the disease, the treatment of huge RHT in an asymptomatic stable patient with ES is still not standardized. Surgical embolectomy was a safe and effective treatment option in this case.

## Author contributions

**Conceptualization:** Ya-Chen Yang.

**Supervision:** Yen-Yu Chen.

**Writing – original draft:** Ya-Chen Yang.

**Writing – review & editing:** Yen-Yu Chen.
